# Multi-Target Joint Detection; Tracking and Classification Based on Marginal GLMB Filter and Belief Function Theory

**DOI:** 10.3390/s20154235

**Published:** 2020-07-29

**Authors:** Jun Liang, Minzhe Li, Zhongliang Jing, Han Pan

**Affiliations:** 1 School of Aeronautics and Astronautics, Shanghai Jiao Tong University, Shanghai 200240, China; liangjuncalt@sina.com (J.L.); zljing@sjtu.edu.cn (Z.J.); hanpan@sjtu.edu.cn (H.P.); 2 China Academy of Launch Vehicle Technology, Beijing 100076, China

**Keywords:** joint detection, tracking and classification, continuous transferable belief model, marginal GLMB filter, multi-model

## Abstract

This paper proposes a new solution to multi-target joint detection, tracking and classification based on labeled random finite set (RFS) and belief function theory. A class dependent multi-model marginal generalized labeled multi-Bernoulli (MGLMB) filter is developed to analytically calculate the multi-target number, state estimates and model probabilities. In addition, a two-level classifier based on continuous transferable belief model (cTBM) is designed for target classification. To make full use of the kinematic characteristics for classification, both the dynamic modes and states are considered in the classifier, the model dependent class beliefs are computed on the continuous state feature subspace corresponding to different dynamic modes and then fused. As a result that the uncertainty about the classes is well described for decision, the classification results are more reasonable and robust. Moreover, as the estimation and classification problems are jointly solved, the tracking and classification performance are both improved. In the simulation, a scenario contains multi-target with miss detection and dense clutter is used. The performance of multi-target detection, tracking and classification is better than traditional methods based on Bayesian classifier or single model. Simulation results are illustrated to demonstrate the effectiveness and superiority of the proposed algorithm.

## 1. Introduction

Multiple target joint detection, tracking and classification is a critical problem in radar system, this problem consists of three subproblems: estimate the number of the targets, estimate their kinematic states and determine their classes. These three subproblems are usually coupled: tracking provides the kinematic features to distinguish the target type; according to the target class, appropriate dynamic models can be chosen for accurate tracking; besides, the detection of the target is the prerequisite of accurate multi-target tracking and classification [1,2,3]. Therefore, multi-target detection, tracking and classification need to be solved jointly.

The traditional joint detection, tracking and classification methods are usually developed based on the Bayesian theory and density estimation in the case of only the position measurements are available. The class probabilities and class dependent multi-target density are calculated based on the Bayesian framework. Recently, the belief function theory has been proven effective in dealing with uncertain information, and many credal models are proposed for target classification and identification, especially the transferable belief model (TBM) proposed by Smets [4,5,6]. Various joint tracking and classification algorithms are also developed based on the TBM. These methods deal with identification of objects as members of predefined classification categories. When only the position measurements are available, the target classes are mainly judged according to the kinematic features, and the proposed solution usually contains the tracker and the classifier. In [7], the dynamic behavior is described using a set of maneuver modes with different acceleration quantity according to the priori. The dynamic state estimates and model probabilities are first calculated using a bank of Kalman filters in parallel, then the beliefs and plausibilities of dynamic behaviors are computed. According to the relationship between dynamic behaviors and the target classes, the classification results are finally obtained. In [8], the Kalman filter is derived within the TBM framework, and the target joint tracking and classification problem is solved within the unified belief theoretical framework. In [9], TBM is employed in the wireless sensor networks, and the terrain information is used to improve the performance. In [10], particle filter is introduced into TBM framework to solve target joint tracking and classification problem in multi-sensor scenario. In [11], a second-order uncertainty model is proposed to describe the uncertain mapping from the dynamic feature space to the target class space, and a practical method based on TBM is provided to calculate the class likelihood under a relaxed dependence assumption. However, due to the discrete nature of such set theoretic uncertain reasoning approaches based on TBM, these algorithms have difficulty in modeling continuous signals. Therefore, the dynamic features such as the state estimates can not be directly used by the classifier. As a result, the accuracy of the classification results is affected.

To overcome such inherent defects, the belief function theory on continuous frame is recently developed by Smets, and the continuous transferable belief model (cTBM) is proposed in [12,13]. In this theory, the basic belief masses allocated on a real value is generalized into belief density on the whole real axis R. The least committed belief functions are first built according to the priori pignistic probability density. Then the basic belief assignment of the target classes is calculated, and the classification results are finally derived using belief function tools. In these articles, the cTBM is also used for target classification, and the advantages compared to the Bayesian methods is also analyzed and illustrated. In [14], the target joint tracking and classification problem with nonlinear trajectories is solved using cTBM and particle filter. As shown in the simulations, the classification results derived based on cTBM are more accurate compared with that of the method based on TBM. However, this approach is difficult to apply to n-dimensional state space $R^{n}$ unless it is independent between different dimensions [13]. Unfortunately, this is usually not satisfied during the dynamic process. In [15], the belief functions are built on $R^{2}$. In [16], a new algorithm is developed when the knowledge of sensors is an α-stable probability density function, the plausibility functions are calculated using a single Gaussian and α-stable model. However, the computational complexities of these methods are high, and it is difficult to extend these approaches to more complicated scenarios. Therefore, in the conventional algorithms, the cTBM based classification results are usually derived based on the velocity or acceleration. Actually, the dynamic characteristics are not fully utilized.

The multi-target joint detection, tracking and classification problem in cluttered environment is much more complex, especially with target number and observation uncertainty. Inaccurate multi-target state estimates and model probabilities calculated under the condition of association error may lead to incorrect classification results. In [17], the multi-target joint tracking and classification problem is solved based on the global nearest neighbor (GNN) approach and TBM. As shown in the simulations, when there exists association error in tracking process, the classification performance is deteriorated. Recently, the random finite set (RFS) theory is developed, and many multi-target tracker are proposed, such as probability hypothesis density (PHD) filter, cardinalized PHD (CPHD) filter and cardinality balanced multi-target multi-Bernoulli (CMeMBer) filter [18,19]. These RFS based trackers are integrated approaches for multi-target joint detection and tracking, and provide the approximated multi-target density with association uncertainty. Compared to traditional approaches [20,21,22,23], the multi-target joint detection, tracking and classification problem is also solved using multi-model PHD/CPHD [1,24,25,26,27,28] and CMeMBer filter [29]. However, due to track information of the RFS based filters can not be obtained directly, these algorithms only calculate the class-dependent multi-target density without the explicit classification results for each target. In [30], the concept of labeled RFS is proposed and the conjugate multi-target state distribution is established. Several multi-target state filters are also derived, such as the generalized labeled multi-Bernoulli (GLMB) filter [31], which provides analytical solutions for multi-target tracking and produce track-valued target state estimates. As demonstrated in the simulations, the performance of the GLMB filter is much better than that of the tracker based on unlabeled RFS. In [32], the target birth intensity are adaptively modeled and introduced to the multi-Bernoulli filter to improve the estimation performance, while the filtering efficiency are maintained. However, the amount of calculation of the GLMB filter increases exponentially with time [33]. In [34], the marginal GLMB (MGLMB) density is proposed to exactly match the posterior multi-target density and the cardinality distribution, this approximate multi-target density is propagated during the estimation process and the MGLMB filter is developed. The performance of MGLMB filter is close to that of the GLMB filter, but the calculation complexity is largely reduced. In [35,36,37], multi-target detection, tracking and classification problems have also been solved using the GLMB filter, these approaches take advantages of the GLMB filter, and explicit class probabilities and state estimates of each target are produced. However, the estimation and classification results are still calculated within Bayesian theory framework.

In this paper, a joint solution is proposed to multi-target detection, tracking and classification based on labeled RFS and belief function theory in the case of only the position measurements are available. A class dependent multi-model MGLMB filter is developed to calculate the multi-target cardinality, states estimates and model probabilities, and a two-level classifier is designed based on the cTBM to identify the target classes using the kinematic data. According to different dynamic models, the class beliefs are computed on corresponding continuous dynamic feature subspace given the state estimates. Then the model-dependent class beliefs are fused to obtain the final classification results. The proposed algorithm has an analytical form, explicit track-valued state estimates and class probabilities of each target are obtained. In addition, as both the target dynamic state estimates and dynamic modes are considered, the designed classifier makes full use of the dynamic characteristics to improve the classification performance. Furthermore, because the uncertainty of the classes is well described using cTBM, and the detection, tracking and classification problems are jointly solved, both the state estimation and classification performance are improved. The particle implementation of the proposed algorithm is also detailed.

This article is organized as follows. An introduction of the cTBM and the MGLMB filter is presented in Section 2. Section 3 proposes a multi-target joint detection, tracking and classification algorithm. The simulation results are illustrated in Section 4. Finally, conclusions are summarized in Section 5.

## 2. Theory Foundation

### 2.1. Classification in the TBM Framework

The transferable belief model aims to represent quantified beliefs based on the belief functions. This model works on two levels: (1) The credal level, in which the beliefs are quantified. (2) The pignistic level, in which the decisions are made [4].

In [8], a joint tracking and classification (JTC) algorithm is proposed in TBM framework. Compared to the probability theory, the likelihood of an hypothesis is equated to the conditional plausibility of the observation given the hypothesis within the TBM. Assume that the $B=\{b_{i}\},i=1,\cdots ,n$ is the set of target behaviors, and $Z_{t}$ is the measurements, then $pl_{i}=pl\left(Z_{t}|b_{i}\right)$ indicates the measurement likelihoods that matched to behavior $b_{i}$. Consider a vacuous a priori on *B*, the posterior belief can be computed using the General Bayesian Theorem (GBT).

(1) $$\begin{array}{c} m^{B}\left(b|Z_{t}\right)=\prod_{i:b_{i}\in b}pl_{i}\prod_{j:b_{j}\notin b}\left(1-pl_{j}\right),\;\forall b\subset B \end{array}$$

Then the class beliefs can be derived according to the relation between the class set *C* and behavior set *B* as

(2) $$\begin{array}{c} m^{C}=\bar{M}\cdot m^{B} \end{array}$$

where $m^{B}$ represents the vector of the basic belief assignments (BBAs), whose elements are corresponding to that in the power set of *B*, and similarly for $m^{C}$. Therefore, within the TBM framework, the beliefs allocated to the elements of the class focal set, which is the power set of the classes, and the explicit class probability is calculated until the decision is made. On contrary, the Bayesian classifier computes the explicit class probabilities based on the measurement likelihood at each time.

### 2.2. Belief Functions on *R* and Least Committed Belief Density

In [12], Smets extended the belief theory on continuous frames and proposed the continuous transferable belief model (cTBM). Compared with TBM, cTBM operates within continuous domain. The basic belief masses generalized into basic belief densities (bbds), and the belief functions can be computed by the integrations of density functions on the specific space.

**Figure 1:**
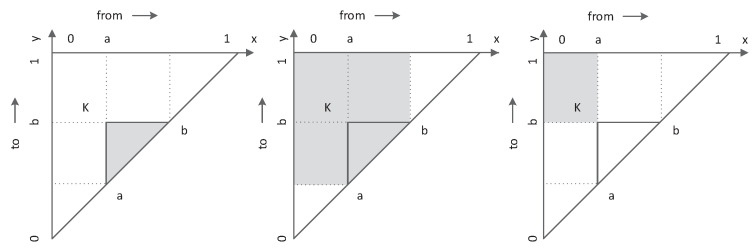
Graphical representations of belief, plausibility and commonality.

Consider the construction of the belief functions on the real space R, and a nonempty interval on the real axis R denoted as [a,b]⊂R,a<b. We assume that masses are only allocated to closed intervals, and the function m:A→R is then a basic belief assignment, where A is the focal set consists of the closed intervals A⊂R. As illustrated in Figure 1, each interval [a,b] corresponds to a point K lies in the triangle, and the point K=(a,b) inside the triangle $T_{\left[0,1\right]}$ uniquely defines the interval $\left[a,b\right]\subset T_{\left[0,1\right]}$. The belief of each point is equal to that of the corresponding interval, $P\left\{\left(x,y\right)=\left(a,b\right)\right\}=m^{A}\left(\left[a,b\right]\right)$. Consequently, the belief, plausibility and commonality of an arbitrary interval [a,b] defined to be the sum of the masses of the intervals [x,y] satisfies {x≥a AND y≤b}, {a≤x≤b OR a≤y≤b} and {x≤a AND y≥b}, respectively.

The commonality denotes the mass that can be assigned on any interval, within, straddling, or outside [a,b]. The pignistic probability is the result of mapping a belief measure to a probability measure. For the singletons within the triangle space, it can be calculated as

(3) $$\begin{array}{c} Betf\left(s\right)=\sum_{s\in A\subset [0,1]}\frac{m(A)}{|a^{*}-a_{*}\left||1-m\left(\emptyset \right)\right|} \end{array}$$

where $a_{*}=inf\left\{a:a\in A\right\}$ and $a^{*}=sup\left\{a:a\in A\right\}$, and $|a^{*}-a_{*}|$ is the interval length. Assume that $f^{T_{\left[0,1\right]}}$ is the basic belief density function, when the number of the focal sets is infinite, this summation is replaced by the integration

(4) $$\begin{array}{c} Betf\left(a\right)=\lim_{\epsilon \to 0}\int_{x=0}^{x=a}\int_{y=a+\epsilon }^{y=1}\frac{f^{T_{\left[0,1\right]}}\left(x,y\right)}{y-x}dxdy \end{array}$$

As a result that there are many bbds which share the same pignistic transformation in Equation (2), the belief density function that maximizes the commonality is defined as the least committed (LC) belief density. As given in [12], the focal interval sets of this density can be represented by a line on the triangle space, which has two properties: (1) For unimodal densities, the line starts at (μ,μ), $\mu =arg\;max_{a}\;etf\left(a\right)$ and $pl^{T}\left(\left[\mu ,\mu \right]\right)=1$. (2) For all symmetrical pignistic densities Betf, the line is straight and centered at μ

(5) $$\begin{array}{c} y=-(x-2\mu )\;-\infty <x<\mu \end{array}$$

The LC belief density φ(a) of interval *A* is

(6) $$\begin{array}{c} \varphi \left(a\right)=-\left(a-\bar{a}\right)\frac{Betf(a)}{da} \end{array}$$

where $\bar{a}$ is defined by the property that: $Betf\left(\bar{a}\right)=Betf\left(a\right)$. Note that $\bar{a}$ is a function of *a*. The plausibility function pl(x) can be computed by the integration of the basic belief density, and the limits indicate the focal intervals have non-empty intersection with *x*, with the property that x≤a≤∞

(7) $$\begin{array}{c} pl\left(x\right)=\left(x-\bar{x}\right)Betf\left(x\right)+\int_{x}^{\infty }\left(1-\frac{d\bar{a}}{da}\right)Betf\left(a\right)da \end{array}$$

Suppose that the pignistic density is Gaussian Betf(x)=N(x;μ,σ), the LC belief density and corresponding plausibility pl(x) are

(8) $$\begin{array}{ccc} \kappa (y) & = & \frac{2y^{2}}{\sqrt{2\pi }}e^{-y^{2}/2} \end{array}$$

(9) $$\begin{array}{ccc} pl(y) & = & \frac{2y}{\sqrt{2\pi }}e^{-y^{2}/2}+erfc\left(y/\sqrt{2}\right) \end{array}$$

where $erfc\left(s\right)=\left(2/\sqrt{\pi }\right)\int_{s}^{\inf}e^{-t^{2}}dt$.

To solve the JTC problem in the cTBM frame, beliefs transfer when new information is received. Let $\Theta =\{\theta_{1},\cdots ,\theta_{n}\}$ is the frame of the target classes, the posterior conditional basic belief assignment $m^{\Theta }\left(A|Z\right)$ of x∈X can be calculated using the priori belief $bel_{\Theta }$ and the plausibility function $pl_{\Theta }\left(\cdot |x\right)$ given x∈X based on the GBT given in (1). Then the a posteriori conditional belief function over θ∈A can be recursively calculated using m(A|Z) and the priori belief $m_{k-1}\left(A\right)$ based on the Dempster’s rule.

(10) $$\begin{array}{c} m_{k}\left(A\right)=m\left(A|Z_{k}\right)⊗m_{k-1}\left(A\right) \end{array}$$

The main difference between TBM and cTBM is that, in TBM the least committed plausibility is computed for the behavior, while in cTBM, the plausibility can be computed using the estimates. In the multi-target tracking scenario, the dynamic states are continuous variables, therefore, the cTBM is more effective than TBM.

### 2.3. Marginal Generalized Labeled Multi-Bernoulli Filter

In [30], Vo et al. introduced the notion of the labeled RFS and constructed conjugate prior. Suppose that the state vector *x* in the space X is augmented with a unique label ℓ∈L, where L is a discrete label space, and X represents the labeled RFS. Let L:L×X→L be the projection L((x,ℓ))=ℓ, L(X) is then the label set of X. The distinct label indicator $\Delta \left(X\right)=\delta_{\left|X\right|}\left(L\left(X\right)\right)$ ensures the distinctness of the labels of X. All the finite subsets of L are denoted by F(L). In a realization of the marginal generalized labeled multi-target state *X*, the labels of two state vectors must be distinct. The distribution of a marginal GLMB RFS is

(11) $$\begin{array}{c} \pi \left(X\right)=\Delta \left(X\right)\sum_{I\in F(L)}\omega^{\left(I\right)}\delta_{I}\left(L\left(X\right)\right){\left[p^{\left(I\right)}\right]}^{X} \end{array}$$

where I∈F(L) denotes the set of track labels, $\omega^{\left(I\right)}$ is the corresponding weight. p(·,ℓ) is the state probability density of track *ℓ*.

As a result that the multi-target conjugate prior constructed by introducing the notion of labeled RFSs is closed under the Chapman–Kolmogorov equation, the marginal GLMB density can be propagated within the Bayesian framework. An analytic solution for the multi-target state estimation is then provided, called marginal GLMB filter [31]. The marginal GLMB filter consists of the following two steps

1. Prediction: suppose that the multi-target prior is a GLMB density of form (12), the predicted multi-target density is then also a GLMB RFS with state space X and label space $L_{+}=L\cup B$ (L∩B=∅), where L and B are the label spaces of the surviving and birth targets. Use the standard inner product notation 〈f,g〉=∫f(x)g(x)dx, the predicted multi-target density can be given by:

(12) $$\begin{array}{c} \pi_{+}\left(X_{+}\right)=\Delta \left(X_{+}\right)\sum_{I\in F(L_{+})}\omega_{+}^{\left(I\right)}\delta_{I}\left(L\left(X_{+}\right)\right){\left[p_{+}^{\left(I\right)}\right]}^{X_{+}} \end{array}$$

where

(13) $$\begin{array}{cc} \omega_{+}^{\left(I\right)} & =\omega_{B}\left(I\cap B\right)\omega_{s}^{\left(I\right)}\left(I\cap L\right) \end{array}$$

(14) $$\begin{array}{cc} p_{+}^{\left(I\right)}\left(x,\ell \right) & =1_{L}\left(\ell \right)p_{s}^{\left(I\right)}\left(x,\ell \right)+\left(1-1_{L}\left(\ell \right)\right)p_{b}\left(x,\ell \right) \end{array}$$

(15) $$\begin{array}{cc} p_{s}^{\left(I\right)}\left(x,\ell \right) & =\frac{\langle p_{s}\left(\cdot ,\ell \right)f\left(x|\cdot ,\ell \right),p^{\left(I\right)}\left(\cdot ,\ell \right)\rangle }{\eta_{s}^{\left(I\right)}\left(\ell \right)} \end{array}$$

(16) $$\begin{array}{cc} \eta_{s}^{\left(I\right)}\left(\ell \right) & =\int \langle p_{s}\left(\cdot ,\ell \right)f\left(x|\cdot ,\ell \right),p^{\left(I\right)}\left(\cdot ,\ell \right)\rangle dx \end{array}$$

(17) $$\begin{array}{cc} \omega_{s}^{\left(I\right)}\left(L\right) & ={\left[\eta_{s}^{\left(I\right)}\right]}^{L}\sum_{I\subset L}1_{I}\left(L\right){\left[q_{s}^{\left(I\right)}\right]}^{I-L}\omega^{\left(I\right)} \end{array}$$

(18) $$\begin{array}{cc} q_{s}^{\left(I\right)}\left(\ell \right) & =\langle q_{s}\left(\cdot ,\ell \right),p^{\left(I\right)}\left(\cdot ,\ell \right)\rangle \end{array}$$

For the predicted multi-target density given a predicted label set *L*, the weight $\omega_{+}^{\left(I\right)}$ is the product of the weight of the new birth and surviving target labels. The surviving target density is predicted using the transition function f(x|·,ℓ).

2. Update: if the multi-target prediction density is a GLMB has the form as (11), the multi-target posterior density is then also a GLMB and has the following form:

$$\begin{array}{c} \pi \left(X|Z\right)=\Delta \left(X\right)\sum_{I\in F(L)}\sum_{\theta \in \Theta }\omega^{\left(I,\theta \right)}\left(Z\right)\delta_{I}\left(L\left(X\right)\right)\times {\left[p^{\left(I,\theta \right)}\left(\cdot |Z\right)\right]}^{X} \end{array}$$

where Θ is the space of mappings θ:L→{0,1,⋯,|Z|}, such that $\theta \left(i\right)=\theta \left(i^{\prime }\right)>0$ implies $i=i^{\prime }$, and

(19) $$\begin{array}{cc} \omega^{\left(I,\theta \right)}\left(Z\right) & \propto {\left[\eta_{Z}^{\left(I,\theta \right)}\right]}^{I} \end{array}$$

(20) $$\begin{array}{cc} p^{\left(I,\theta \right)}\left(x,\ell |Z\right) & =\frac{p^{\left(I\right)}\left(x,\ell \right)\psi_{Z}\left(x,\ell ;\theta \right)}{\eta_{Z}^{\left(I,\theta \right)}\left(\ell \right)} \end{array}$$

(21) $$\begin{array}{cc} \eta_{Z}^{\left(I,\theta \right)}\left(\ell \right) & =\langle p^{\left(I\right)}\left(\cdot ,\ell \right),\psi_{Z}\left(\cdot ,\ell ;\theta \right)\rangle \\ \psi_{Z}\left(x,\ell ;\theta \right) & =\delta_{0}\left(\theta \left(\ell \right)\right)\left(1-p_{d}\left(x,\ell \right)\right) \end{array}$$

(22) $$\begin{array}{cc} \; & +\left(1-\delta_{0}\left(\theta \left(\ell \right)\right)\right)\frac{p_{d}\left(x,\ell \right)g\left(z_{\theta (\ell )}|x,\ell \right)}{\kappa (z_{\left(\theta (\ell )\right)})} \end{array}$$

In the update step, the posterior target density $p^{\left(I,\theta \right)}\left(x,\ell |Z\right)$ given labeled *ℓ* is explicitly calculated using the predicted multi-target density via Bayes rule with likelihood function $\psi_{Z}$. The updated weight $\omega^{\left(I,\theta \right)}\left(Z\right)$ is proportional to the prior weight scaled by the product ${\left[\eta_{Z}^{\left(I,\theta \right)}\right]}^{I}$ of single target normalizing constants. Here, $p_{d}\left(x,\ell \right)$ is the detection probability of track *ℓ*, g(z|x,ℓ) is the likelihood function for the measurement *z* and κ(·) is the intensity of Poisson clutter.

Compute the marginalized GLMB density to exactly match the posterior multi-target density and cardinality distribution of (18), the approximate multi-target density is

(23) $$\begin{array}{c} \hat{\pi }\left(X\right)=\Delta \left(X\right)\sum_{I\in F(L)}\delta_{I}\left(L\left(X\right)\right)w^{\left(I\right)}{\left[p^{\left(I\right)}\right]}^{X} \end{array}$$

where

(24) $$\begin{array}{cc} w^{\left(I\right)} & =\sum_{\theta \in \Theta (I)}w^{\left(I,\theta \right)}\\ p^{\left(I\right)}\left(x,\ell \right) & =1_{I}\left(\ell \right)\frac{1}{w^{\left(I\right)}}\sum_{\theta \in \Theta (I)}w^{\left(I,\theta \right)}p^{\left(I,\theta \right)}\left(x,\ell \right) \end{array}$$

As given in (23), a principled GLMB $\hat{\pi }\left(X\right)$ is constructed approximation to the posterior density π(X), which results in a marginalization over the association histories. As a result that the number of components propagated in the Bayesian recursion is drastically reduced, the computational complexity is largely reduced.

## 3. Multi-Target Joint Detection, Tracking and Classification Algorithm

This section first presents the mathematical formulation of the problem, then proposes the multi-target joint detection, tracking and classification algorithm based on the labeled RFS and cTBM. The particle implementation of the proposed algorithm is also detailed.

### 3.1. Problem Formulation

Assume that the class of each target is a time-invariant attribute that takes values from a discrete set $C=\{c_{1},c_{2},\cdots ,c_{n}\}$, and the class-dependent dynamic model set is $M_{c}=\left\{o_{1},o_{2},\cdots ,o_{m}\right\}$. These models transfer given an underlying Markov process with class dependent model transition probability $f(o|o^{\prime },c)$. The target dynamic and measurement equations at time *k* are

(25) $$\begin{array}{ccc} x_{k} & = & f_{k|k-1,o}\left(\cdot |x_{k-1}\right)+w_{k} \end{array}$$

(26) $$\begin{array}{ccc} z_{k} & = & h_{k}\left(\cdot |x_{k}\right)+v_{k} \end{array}$$

where $f_{k|k-1,o}\left(\cdot \right)$ is the model-dependent dynamic equation, and $h_{k}$ is the observation function of the targets. $f_{k|k-1,o}\left(\cdot \right)$ and $h_{k}\left(\cdot \right)$ are possibly nonlinear, and $w_{k}$ and $v_{k}$ are uncorrelated Gaussian process and measurement noise.

In multi-target scenario, each target appears and disappears randomly with the birth probability $p_{b,k}$ and the survive probability $p_{s,k}$. At time *k*, $X_{k}=\left\{x_{k,1},x_{k,2},\cdots ,x_{k,n}\right\}$ is the multi-target state set with time varying target numbers, and $Z_{k}=\left\{z_{k,1},z_{k,2},\cdots ,z_{k,m}\right\}$ is the observation set which consists of measurements from targets and clutter with the detection probability $p_{d,k}\left(x_{k}\right)$ and clutter density $c_{k}$.

### 3.2. Multi-Target Joint Detection, Tracking and Classification Algorithm Based on Labeled RFS and cTBM

**Figure 2:**
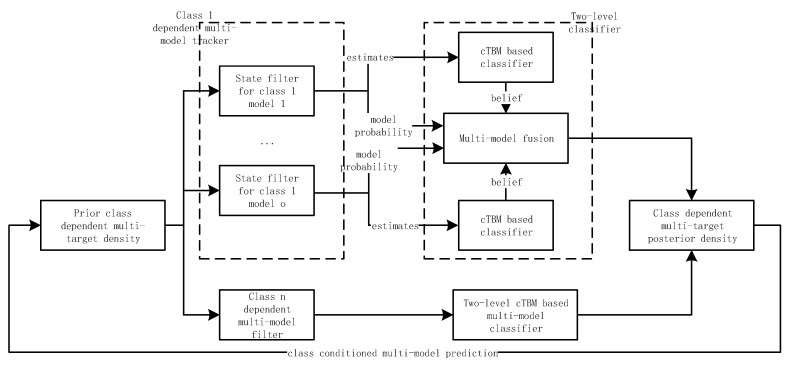
Algorithm flow diagram.

The proposed algorithm contains the tracker and classifier. Starting with prior multi-target density, the class dependent multi-target posterior density is first calculated using the multi-model marginal GLMB filter. Then, the classification results are derived using the two-level classifier based on cTBM. In the classifier, the model dependent class basic belief assignments (BBAs) are computed using the state estimates, then the beliefs are fused to derive the final classification results. The algorithm diagram is shown in Figure 2. The particle implementation is also provided in this section.

A. Class dependent multi-model MGLMB filter

(1) Prediction: Assume at time k−1, the class dependent posterior multi-target density is

(27) $$\begin{array}{c} \pi_{k-1}\left(X\right)=\Delta \left(X\right)\sum_{I\in L(X)}\sum_{c\in C}\omega_{k-1}^{\left(I\right)}\delta_{I}\left(L\left(X\right)\right){\left[p_{k-1,\ell }^{\left(I\right)}\left(\cdot ,o|c\right)\right]}^{X} \end{array}$$

where ${\left[p_{k-1,\ell }^{\left(I\right)}\left(\cdot ,o^{\prime }|c\right)\right]}^{X}$ is the model dependent multi-target target density given the classes. The density of each target *ℓ* can be further expressed as $p_{k-1,\ell }\left(x^{\prime },o^{\prime }|c\right)$. At time *k*, the predicted multi-target density is

(28) $$\begin{array}{c} \pi_{k+}\left(X_{+}\right)=\Delta \left(X_{+}\right)\;\;\sum_{I\in L(X_{+})}\sum_{c\in C}\omega_{k+}^{\left(I\right)}\delta_{I}\left(L\left(X\right)\right){\left[p_{k+,\ell }^{\left(I\right)}\left(\cdot ,o|c\right)\right]}^{X_{+}} \end{array}$$

where

(29) $$\begin{array}{cc} \omega_{k+}^{\left(I\right)} & =\omega_{B}\left(I\cap B\right)\omega_{s}^{\left(I\right)}\left(I\cap L\right) \end{array}$$

(30) $$\begin{array}{cc} p_{k+,\ell }^{\left(I\right)}\left(x,o|c\right) & =\sum_{o\in M_{c}}p_{k+}^{\left(I\right)}\left(o|c\right)\frac{\langle p_{s}^{\left(I\right)}\left(x^{\prime }\right)f\left(x|x^{\prime },o^{\prime },c\right),p_{k-1,\ell }^{\left(I\right)}\left(x^{\prime }|o^{\prime },c\right)\rangle }{\eta_{s}^{\left(I\right)}\left(\ell \right)} \end{array}$$

(31) $$\begin{array}{cc} \eta_{s}^{\left(I\right)}\left(\ell \right) & =\sum_{o^{\prime }\in M_{c}}p_{k-1,\ell }^{\left(I\right)}\left(o^{\prime }|c\right)\int p_{s,\ell }\left(x^{\prime }\right)p_{k-1,\ell }^{\left(I\right)}\left(x^{\prime }|o^{\prime },c\right)dx^{\prime } \end{array}$$

(32) $$\begin{array}{cc} p_{k+,\ell }^{\left(I\right)}\left(o|c\right) & =\sum_{o^{\prime }\in M_{c}}p\left(o|o^{\prime },c\right)p_{k-1,\ell }^{\left(I\right)}\left(o^{\prime }|c\right) \end{array}$$

In these equations, L and B denote the label spaces of the surviving targets and the new birth targets, respectively, $f(x|x^{\prime },o^{\prime },c)$ is the model dependent dynamic equation, $p(o|o^{\prime },c)$ is the class dependent model transition probability and $p_{s,\ell }$ is the surviving probability of track *ℓ*.

(2) Update: Assume that the predicted multi-target density is represented as

(33) $$\begin{array}{c} \pi_{k+}\left(X_{+}\right)=\Delta \left(X_{+}\right)\;\;\sum_{I\in L(X_{+})}\sum_{c\in C}\omega_{k+}^{\left(I\right)}\delta_{I}\left(L\left(X_{+}\right)\right){\left[p_{k+,\ell }^{\left(I\right)}\left(\cdot ,o|c\right)\right]}^{X_{+}} \end{array}$$

when the measurement set *Z* is received, the multi-target posterior density is

(34) $$\begin{array}{c} \pi_{k}\left(X\right)=\Delta \left(X\right)\sum_{\xi \in \Xi }\sum_{c\in C}\omega_{k}^{\left(\xi \right)}\delta_{I}\left(L\left(X\right)\right){\left[p_{k,\ell }^{\left(\xi \right)}\left(\cdot ,o|c\right)\right]}^{X} \end{array}$$

where ξ∈Ξ represents $\left(I,\theta \right)\in F\left(L_{+}\right)\times \Theta$, Θ is the space of mappings θ:L→{0,1,⋯,|Z|}, such that $\theta \left(i\right)=\theta \left(i^{\prime }\right)>0$ implies $i=i^{\prime }$, and

(35) $$\begin{array}{cc} \omega_{c,k}^{\left(\xi \right)}\left(Z\right) & \propto \omega_{c,k+}^{\left(I\right)}{\left[\eta_{Z}^{\left(\xi \right)}\right]}^{I} \end{array}$$

(36) $$\begin{array}{cc} p_{k,\ell }^{\left(\xi \right)}\left(x|o,c\right) & =\frac{p_{k+,\ell }^{\left(I\right)}\left(x|o,c\right)\psi_{Z}^{\left(\xi \right)}\left(x|o,c\right)}{\eta_{Z}^{\left(\xi \right)}\left(\ell |o,c\right)} \end{array}$$

(37) $$\begin{array}{cc} \eta_{Z}^{\left(\xi \right)}\left(\ell |o,c\right) & =\langle p_{k+,\ell }^{\left(I\right)}\left(x|o,c\right),\psi_{Z}^{\left(\xi \right)}\left(x|o,c\right)\rangle \end{array}$$

(38) $$\begin{array}{cc} \psi_{Z}^{\left(\xi \right)}\left(x|o,c\right) & =\delta_{0}\left(\theta \left(\ell \right)\right)q_{d}\left(x|o,c\right) \end{array}$$

(39) $$\begin{array}{cc} \; & +\left(1-\delta_{0}\left(\theta \left(\ell \right)\right)\right)\frac{p_{d}\left(x\right)g\left(z_{\theta (\ell )}|x\right)}{\kappa (z_{\theta (\ell )})}\; \end{array}$$

(40) $$\begin{array}{cc} p_{k,\ell }^{\left(\xi \right)}\left(o|c\right) & =\frac{p_{k+,\ell }^{\left(I\right)}\left(o|c\right)\eta_{Z}^{\left(\xi \right)}\left(\ell |o,c\right)}{\eta_{Z}^{\left(\xi \right)}\left(\ell |c\right)} \end{array}$$

(41) $$\begin{array}{cc} \eta_{Z}^{\left(\xi \right)}\left(\ell |c\right) & =\sum_{o\in M_{c}}p_{k+,\ell }^{\left(I\right)}\left(o|c\right)\eta_{Z}^{\left(\xi \right)}\left(\ell |o,c\right) \end{array}$$

In these equations, $\delta_{0}\left(\theta \left(\ell \right)\right)$ indicates whether there is a measurement associated with the target, $g(z_{\theta (\ell )}|x)$ is the likelihood function and $p_{d}$ is the detection probability of the target. ${\left[p_{k,\ell }^{\left(\xi \right)}\left(\cdot ,o|c\right)\right]}^{X}$ is the product of multiple target densities $p_{k,\ell }^{\left(\xi \right)}\left(\cdot ,o|c\right)$ given predicted labels *I* and association map θ. The class dependent marginal GLMB density that matches the class dependent posterior multi-target density and the cardinality distribution is

(42) $$\begin{array}{c} \hat{\pi }\left(X\right)=\Delta \left(X\right)\sum_{I\in F(L)}\sum_{c\in C}\omega_{k}^{\left(I\right)}\delta_{I}\left(L\left(X\right)\right){\left[p_{k,\ell }^{\left(I\right)}\left(\cdot ,o|c\right)\right]}^{X} \end{array}$$

where

(43) $$\begin{array}{cc} \omega_{k}^{\left(I\right)} & =\sum_{\theta \in \Theta }\omega_{k}^{\left(\xi \right)} \end{array}$$

(44) $$\begin{array}{cc} p_{k,\ell }^{\left(I\right)}\left(x,o|c\right) & =1_{I}\left(\ell \right)\frac{1}{\omega_{k}^{\left(I\right)}}\sum_{\theta \in \Theta }\omega_{k}^{\left(\xi \right)}p_{k,\ell }^{\left(\xi \right)}\left(x,o|c\right) \end{array}$$

and the target number is

(45) $$\begin{array}{c} N=\sum_{I\in F(L)}n\times \omega_{k}^{\left(I\right)} \end{array}$$

where *n* is the number of the targets in label set L.

B. Classification based on cTBM

The dynamic features are associated with both dynamic states and motion modes. For example, the target shows a higher maneuverability when executing a sharp turn than fling straight at the same speed; the cruising speed of fighters may exceed sonic velocity, which is much faster than most normal planes. Obviously, the classification based on either dynamic states or motion modes is partial. Therefore, a two-level classifier is designed here, and both of these two factors are considered. The proposed classifier consists of the following components.

(1) Classification on the state feature subspace

As a result that the dynamic states of different dimensions are usually considered as joint, such as the speed and acceleration, the calculation of the beliefs based on cTBM in the framework of a one-dimensional continuous state feature space is difficult to be directly extended to multi-dimensional feature space. Assume that $x_{k,o,\ell }$ is the posterior state estimate of track ℓ∈I given the model *o*. In the proposed algorithm, one-dimensional state feature subspace is firstly chosen corresponding to the dynamic models, and the conditional LC plausibility $pl(x_{k,o,\ell }|c_{i})$ is calculated using the state sub-vector ${\tilde{x}}_{k,o,\ell }\in R$. Then the beliefs are fused to derive the final classification results. Suppose that $Betf({\tilde{x}}_{k,o,\ell }|c_{i},o_{j})$ is the prior pignistic density function of target class $c_{i}$ and model $o_{j}$, then the LC plausibility can be computed as

(46) $$\begin{array}{ccc} pl({\tilde{x}}_{k,o,\ell }|c_{i},o_{j}) & = & \left({\tilde{x}}_{k,o,\ell }-\phi \left({\tilde{x}}_{k,o,\ell }\right)\right)Betf\left({\tilde{x}}_{k,o,\ell }|c_{i},o_{j}\right) \end{array}$$

(47) $$\begin{array}{cc} + & \int_{{\tilde{x}}_{k,o,\ell }}^{\infty }\left(1-\frac{d\phi (a)}{da}\right)Betf\left(a|c_{i},o_{j}\right)da \end{array}$$

where $\phi ({\tilde{x}}_{k,o,\ell })$ satisfies

(48) $$\begin{array}{c} Betf\left(\phi \left({\tilde{x}}_{k,o,\ell }\right)|c_{i},o_{j}\right)=Betf\left({\tilde{x}}_{k,o,\ell }|c_{i},o_{j}\right) \end{array}$$

and the state sub-vector ${\tilde{x}}_{k,o,\ell }$ is selectable according to different modes. For example, velocity or acceleration are chosen for classifying corresponding to CV or CA models, respectively. Using the Generalized Bayesian Theorem (GBT), the model dependent BBAs of the classes are

(49) $$\begin{array}{c} m\left(C|{\tilde{x}}_{k,o,\ell },o_{j}\right)=\prod_{c_{i}\in C}pl\left({\tilde{x}}_{k,o,\ell }|c_{i},o_{j}\right)\times \prod_{c_{i}\in \bar{C}}\left[1-pl\left({\tilde{x}}_{k,o,\ell }|c_{i},o_{j}\right)\right] \end{array}$$

(2) Classification with multi-model

Let (C,X,O) indicates the credibility space, where X and O are the state and model frames, respectively, while the beliefs allocated to the elements of C. Combine the model dependent beliefs $m(C|{\tilde{x}}_{k,o,\ell },o)$ calculated before, the final class BBAs of target *ℓ* is

(50) $$\begin{array}{c} m\left(C|x_{k,\ell }\right)=\sum_{o_{j}\in M_{c}}m\left(C|{\tilde{x}}_{k,o,\ell },o_{j}\right)m\left(o_{j}\right) \end{array}$$

where $m(o_{j})$ is the confidence allocated on the models, which is equal to the model probability [38]. This equation can also be represented using the matrix equation $m^{C}=\bar{M}m^{o}$, where $m^{C}=\left[m^{c_{1}},m^{c_{2}},\cdots ,m^{c_{n}}\right]$ is the vector of BBAs, C is the power set of the class, and $m^{o}=\left[m^{o_{1}},m^{o_{2}},\cdots ,m^{o_{m}}\right]$ is the vector of model belief masses. The dimension of these two vectors are not need to be equal, this conforms the situation that the dynamic models and the classes are not in one-to-one correspondence. Each column of the matrix $\bar{M}$ is the model dependent BBAs of class focal given the selectable state sub-vector ${\tilde{x}}_{k,o,\ell }$.

(3) Time recursion using Generalized Bayesian Theorem

Combine the conditional beliefs with prior information using (9), the posterior class beliefs $m_{k}\left(C|x_{k,o,\ell }\right)$ at time *k* can be calculated. Then, the pignistic class probabilities are

(51) $$\begin{array}{c} BetP_{k}\left\{c_{i}|x_{k,o,\ell }\right\}=\sum_{c_{i}\in C}\frac{1}{\left|C\right|}\frac{m_{k}\left(C|x_{k,o,\ell }\right)}{1-m_{k}\left(\emptyset |x_{k,o,\ell }\right)} \end{array}$$

The proposed MGLMB-cTBM algorithm for multi-target joint detection, tracking and classification based on labeled RFS and belief function theory is summarized as follows:
**Algorithm 1:** MGLMB-cTBM algorithm for multi-target joint detection, tracking and classificationInitial the prior conditional pignistic density function of the dynamic states.(1) Predict the multi-target density $\pi_{k+}\left(X_{+}\right)$, and model dependent density $p_{k+,\ell }^{\left(I\right)}\left(x,o|c\right)$ and model probability $p_{k+,\ell }^{\left(I\right)}\left(o|c\right)$ of each target using (28)–(32).(2) Update the multi-target density $\pi_{k}\left(X\right)$, model dependent density $p_{k,\ell }^{\left(\xi \right)}\left(x|o,c\right)$ and model probability $p_{k,\ell }^{\left(\xi \right)}\left(o|c\right)$ using (34)–(41).(3) Compute the marginal GLMB density $\hat{\pi }\left(X\right)$ using (42)–(44).(4) For different dynamic models, chose the state estimate sub-vector ${\tilde{x}}_{k,o,\ell }\in R$ to calculate the conditional LC plausibility $pl({\tilde{x}}_{k,o,\ell }|c_{i},o_{j})$, and then compute the model dependent beliefs $m(C|{\tilde{x}}_{k,o,\ell },o_{j})$ using (46)–(48).(5) Combine the model dependent beliefs to compute the final BBAs of the class focal $m(C|x_{k,\ell })$ as (49).(6) Merge the beliefs with the priori using GBT, and then go to step 1.

**Analysis and discussion**: 1. In this paper, an analytical algorithm is proposed to multi-target joint detection, tracking and classification in radar system. In this application, the target is embodied in the form of points. For the point targets, all the information about the target can be represented using the state and category. Therefore, both the observations and the dynamic information can be directly denoted using the measurement and state vectors. The posterior state and class probabilities can be analytically updated, and the classification results can be explicitly derived based on the dynamic states and the prior distribution of the class pignistic probability obtained directly from statistical data. Therefore, the derived results are Bayesian optimal. Compared to the traditional methods in [28,36], the dependence between estimation and classification is considered in this paper. Multi-target density is predicted using the class dependent multi-model, and the classifier computes the target class according to the both the dynamic models and estimates. The relation between multi-target class and state estimation is reflected in both the prediction and update process of the proposed algorithm. As given in (36)–(42), the mapping weights are updated based on the class dependent multi-model likelihood, therefore, the target existing probability (detection) is derived related to the target class in (45). As given in (43)–(45), the target density is the sum of weighed density updated given the target classes, therefore, the multi-target tracking result is also related to target classes. Therefore, according to the target class, appropriate models are used for improve the detection and tracking performance. Furthermore, more accurate estimation results lead to the improvement of the classification and overall performance.

2. In the cTBM based classifier, the classification results are explicitly derived based on the dynamic state estimates and the prior distribution of the class pignistic probability obtained directly from statistical data. Therefore, the classification results provided by the classifier are Bayesian optimal. During the solution process, the LC plausibilities of target classes are first computed, then the BBAs of the class focal sets are derived. The target class focal set is the power set of the target classes. The number of the focal elements in the set is $2^{n}$, where n is the number of possible class in the frame of discernment. The beliefs allocated to the elements of the class focal set, which is the power set of the classes. For example, when the two categories c1 and c2 can not be completely distinguished, the belief mass is assigned to the elements {c1c2}. The belief assignments are combine with the prior beliefs using the GBT, and the explicit class probability is calculated until the decision is made. On contrary, the Bayesian classifier computes the class probabilities based on the measurement likelihood at each time. Moreover, compared with the TBM based methods, cTBM based classifier can derive the classification results directly using the dynamic estimates. When there is no effective information contained in the dynamic model (the target moves straight with the same velocity all the time), the class can still be judged according to the dynamic estimates. In addition, the models are selected according to the dynamic modes. By contrast, for the TBM, the classification result is derived based on the dynamic modes, which is actually an interval in the state space, such as the accelerate a∈[−g,+g]. Therefore, the uncertainty about the class is described more precisely and the cTBM based classifier is better than the traditional approaches. Furthermore, as the number of model is largely reduced, the computational complexity is reduced and the model competition is avoided.

3. The computation complexity of the proposed MGLMB-cTBM algorithm is generally higher than that of the standard GLMB filter. Assume that P=max|L(X)| is the maximum number of the targets, and M=|Z| is the maximum number of the measurements, as given in [33], the computational complexity of GLMB filter is $O(P^{2}M)$, i.e., quadratic in the number of hypothesized labels and linear in the number of measurements. The computational complexity of marginal GLMB filter is the same as that of the GLMB filter in the worst case. In practical applications, for the classifier of the proposed MGLMB-cTBM algorithm, the integral calculation in (46) can be calculated previously based on the prior pignistic probability density, and the belief assignments given the estimates can be quickly obtained by looking up the table. Therefore, the main difference of the computational complexity between the MGLMB-cTBM and MGLMB filter lies in the estimation process given different classes and models, which involves predicting and updating the target state according to the class dependent multi-model. Assume that *C* and *O* are the maximum number of the target class focal sets and models, the computational complexity of MGLMB-cTBM is $O({\left(COP\right)}^{2}M)$.

4. With the application of optoelectronic technology and the improvement of radar resolution, the target morphological information can also be obtained, such targets are treated as the image targets and extended targets, respectively. In [39,40,41], the graph neural network (GNN) is used for multiple image targets tracking. The graph nodes are composed of all the targets in all frames. The attributes of the nodes are composed of apparent features and geometric features (position and shape) obtained through training, and the distance of the nodes are defined using Euclidean distance, etc. There are connections between nodes across frames, and the weights of the edges are setting based on the defined distance metric. The association and tracking results are derived through recursively updating the weights of the edges and the attributes of the nodes. In [42,43], convolutional neural networks (CNN) and long short-term memory (LSTM) are used to obtain the kinematic and morphological characteristics of the target, and the dynamic states and association relationships of the targets are calculated using the GNN. In [44], the importance of learned reID features for multi-object tracking is shown. These proposed GNN-based methods can also be used for multiple extended targets tracking. Moreover, in the scenario when the attribute measurements are available, such as the signal features, the belief of the attribute measurements can be fused with the beliefs calculated using the dynamic estimates based on the D-S evidence theory. Relevant works can be done in the future.

### 3.3. The Particle Implementation

(1) Prediction: As given in (28), the predicted multi-target density $\pi_{k+}\left(X_{+}\right)$ consists of the model dependent density of each target. Using the particle description, the density $p_{k+,\ell }^{\left(I\right)}\left(x,o|c\right)$ of track *ℓ* can be denoted as ${\left\{x_{k+,o,\ell }^{\left(n\right)},\omega_{k+,o,\ell }^{\left(n\right)}\right\}}_{n=1}^{N_{k+,o,\ell }}$, where $x_{k+,o,\ell }$ and $\omega_{k+,o,\ell }$ are the predicted state and weight that calculated using the state transition matrix $F_{o}$ and surviving probability $p_{s}^{\ell }$

(52) $$\begin{array}{ccc} x_{k+,o,\ell }^{\left(n\right)} & = & F_{o}x_{k-1,o,\ell }^{\left(n\right)} \end{array}$$

(53) $$\begin{array}{ccc} \omega_{k+,o,\ell }^{\left(n\right)} & = & p_{s}^{\ell }\omega_{k-1,o,\ell }^{\left(n\right)} \end{array}$$

The predicted density of new birth target *ℓ* can be indicated using particles ${\left\{x_{b,o,\ell }^{\left(n\right)},\omega_{b,o,\ell }^{\left(n\right)}\right\}}_{n=1}^{N_{b,o,\ell }}$, where the state $x_{b,o,\ell }^{\left(n\right)}$ and weight $\omega_{b,o,\ell }^{\left(n\right)}$ conform the prior.

(2) Update: The posterior density $p_{k,\ell }^{\left(\xi \right)}\left(x|o,c\right)$ of target *ℓ* can be indicated using particles ${\left\{x_{k,o,\ell }^{\left(n\right)},\omega_{k,o,\ell }^{\left(n\right)}\right\}}_{n=1}^{N_{k,o,\ell }}$, where the weights

(54) $$\begin{array}{c} \omega_{k,o,\ell }^{\left(n\right)}=\omega_{k+,o,\ell }^{\left(n\right)}\left(\delta_{0}\left(\theta \left(\ell \right)\right)\left(1-p_{d}\right)+\left(1-\delta_{0}\left(\theta \left(\ell \right)\right)\right)\frac{p_{d}q\left(x_{k_{+},o,\ell }^{\left(n\right)}\right)\delta \left(x_{k}^{\left(n\right)}-x_{k+}^{\left(n\right)},o_{k}^{\left(n\right)}-o_{k+}^{\left(n\right)}\right)}{\kappa \left(z_{\theta (\ell )}\right)+\sum_{o\in M_{c}}\sum_{n=1}^{N_{k+,o,\ell }}q\left(x_{k_{+},o,\ell }^{\left(n\right)}\right)}\right) \end{array}$$

(55) $$\begin{array}{c} q\left(x_{k_{+},o,\ell }^{\left(n\right)}\right)=N\left(z_{\theta (\ell )};H_{k}x_{k}^{\left(n\right)},R_{k}\right) \end{array}$$

In these equations, the term $x_{k,o,\ell }^{\left(n\right)}$ and $\omega_{k,o,\ell }^{\left(n\right)}$ are the posterior state particles and associated weights, $\delta_{0}\left(\theta \left(\ell \right)\right)$ indicates whether there is a measurement associated with the target, $q(x_{k+,o,\ell }^{\left(n\right)})$ is the likelihood function, and $p_{d}$ is the detection probability of the target. The dynamic model probabilities are $p_{c,k}\left(o\right)=\sum_{n=1}^{N_{k+,o,\ell }}\omega_{c,k,o,\ell }^{\left(n\right)}$.

(3) Resample: To avoid particle impoverishment and prevent exponential growth of the particle number, a resample procedure is performed, and then the derived particles have equal weights

(56) $$\begin{array}{c} {\bar{\omega }}_{k,o,\ell }^{\left(n\right)}=\frac{\omega_{k,o,\ell }^{\left(n\right)}}{\sum_{o\in M_{c}}\sum_{n=1}^{N_{k+,o,\ell }}\omega_{k,o,\ell }^{\left(n\right)}} \end{array}$$

(4) Compute the BBAs: For each particle $\left\{x_{k,o,\ell }^{\left(n\right)},\omega_{k,o,\ell }^{\left(n\right)}\right\}$, the state sub-vector ${\tilde{x}}_{k,o,\ell }$ is selectable according to different modes $o_{j}$. Suppose that $Betf({\tilde{x}}_{k,o,\ell }^{\left(n\right)}|c_{i},o_{j})$ is the prior pignistic density function of target class $c_{i}$ and model $o_{j}$. Then, the LC plausibility and BBAs can be computed using the state estimates of each particle as

(57) $$\begin{array}{ccc} pl({\tilde{x}}_{k,o,\ell }^{\left(n\right)}|c_{i},o_{j}) & = & \left({\tilde{x}}_{k,o,\ell }^{\left(n\right)}-\phi \left({\tilde{x}}_{k,o,\ell }^{\left(n\right)}\right)\right)Betf\left({\tilde{x}}_{k,o,\ell }^{\left(n\right)}|c_{i},o_{j}\right) \end{array}$$

(58) $$\begin{array}{cc} + & \int_{{\tilde{x}}_{k,o,\ell }^{\left(n\right)}}^{\infty }\left(1-\frac{d\phi (a)}{da}\right)Betf\left(a|c_{i},o_{j}\right)da \end{array}$$

(59) $$\begin{array}{c} m\left(C|{\tilde{x}}_{k,o,\ell }^{\left(n\right)},o_{j}\right)=\prod_{c_{i}\in C}pl\left({\tilde{x}}_{k,o,\ell }^{\left(n\right)}|c_{i},o_{j}\right)\times \prod_{c_{i}\in \bar{C}}\left[1-pl\left({\tilde{x}}_{k,o,\ell }^{\left(n\right)}|c_{i},o_{j}\right)\right] \end{array}$$

Combine all of the BBAs from each of the particles for model *o* using D-S theory [14], the fused model conditioned BBAs can be calculated as

(60) $$\begin{array}{c} m_{1⊕2}\left(C|{\tilde{x}}_{k,o,\ell },o_{j}\right)=\frac{\sum_{B\cap C}m\left(C|{\tilde{x}}_{k,o,\ell }^{1},o_{j}\right)m\left(C|{\tilde{x}}_{k,o,\ell }^{2},o_{j}\right)}{1-\sum_{B\cap C=\emptyset }m\left(C|{\tilde{x}}_{k,o,\ell }^{1},o_{j}\right)m\left(C|{\tilde{x}}_{k,o,\ell }^{2},o_{j}\right)} \end{array}$$

(5) Classification with multi-model: Combine the model dependent beliefs $m(C|{\tilde{x}}_{k,o,\ell },o)$, the final class BBAs of target *ℓ* is

(61) $$\begin{array}{c} m\left(C|x_{k,\ell }\right)=\sum_{o_{j}\in M_{c}}m\left(C|{\tilde{x}}_{k,o,\ell },o_{j}\right)m\left(o_{j}\right) \end{array}$$

Fuse the conditional beliefs with prior information to compute the posterior class beliefs $m_{k}\left(C|x_{k,o,\ell }\right)$. Then, the pignistic class probabilities are

(62) $$\begin{array}{c} BetP_{k}\left\{c_{i}|x_{k,o,\ell }\right\}=\sum_{c_{i}\in C}\frac{1}{\left|C\right|}\frac{m_{k}\left(C|x_{k,o,\ell }\right)}{1-m_{k}\left(\emptyset |x_{k,o,\ell }\right)} \end{array}$$

At last, proportionally correct the weights of the particles belong to the class $c_{i}$ conform that $\sum_{o\in M_{c}}\omega_{k,o,\ell }^{\left(n\right)}=BetP_{k}\left\{c_{i}|x_{k,o,\ell }\right\}$.

The implementation can be realized using the open source tools (http://ba-tuong.vo-au.com/codes.html), and the pseudo-code of the MGLMB-cTBM algorithm is given as follows:
**Algorithm 2:** Pseudo-code of the proposed MGLMB-cTBM algorithm1: **function** MGLMB-cTBM algorithm 2: Input ${\left\{x_{k+,o,\ell }^{\left(n\right)},\omega_{k+,o,\ell }^{\left(n\right)}\right\}}_{n=1}^{N_{k+,o,\ell }}$ given different classes and models, input the measurements $Z_{k}$.3: Generate the k-shortest path to calculate k-best births and surviving hypotheses, predict the target state $x_{k+,o,\ell }^{\left(n\right)}$ and $\omega_{k+,o,\ell }^{\left(n\right)}$ using (52) and (53), add the birth particles ${\left\{x_{b,o,\ell }^{\left(n\right)},\omega_{b,o,\ell }^{\left(n\right)}\right\}}_{n=1}^{N_{b,o,\ell }}$.5: **for** $i=1,\cdots ,|Z_{k}|$6: Compute the likelihood $q(z_{i}|x_{k_{+},o,\ell }^{\left(n\right)})$ for each measurement $z_{i}\in Z_{k}$ using (55).7: **end for**8: Calculate m-best assignment hypotheses/components using murty’s algorithm according to the likelihood $q(z_{i}|x_{k}^{\left(n\right)})$.9: Update the weights $\omega_{k,o,\ell }^{\left(n\right)}$ using (54).10: Resample.11: Look up the table and obtain the LC plausibility $pl({\tilde{x}}_{k,o,\ell }^{\left(n\right)}|c_{i},o_{j})$ and BBAs $m(C|{\tilde{x}}_{k,o,\ell }^{\left(n\right)},o_{j})$ according to the estimates $x_{k+,o,\ell }^{\left(n\right)}$ of particles. Compute the dynamic model probability $p_{k}\left(o\right)=\sum_{n=1}^{N_{k+,o,\ell }}\omega_{k,o,\ell }^{\left(n\right)}$.12: Combine the BBAs for model $m_{1⊕2}\left(C|{\tilde{x}}_{k,o,\ell },o_{j}\right)$ using (59).13: Combine the model dependent beliefs $m(C|{\tilde{x}}_{k,o,\ell },o)$, and then merge the beliefs with the priori using GBT to derive the final class BBAs of target *ℓ*. Then go to 1.**end function**

## 4. Simulations

**Figure 3:**
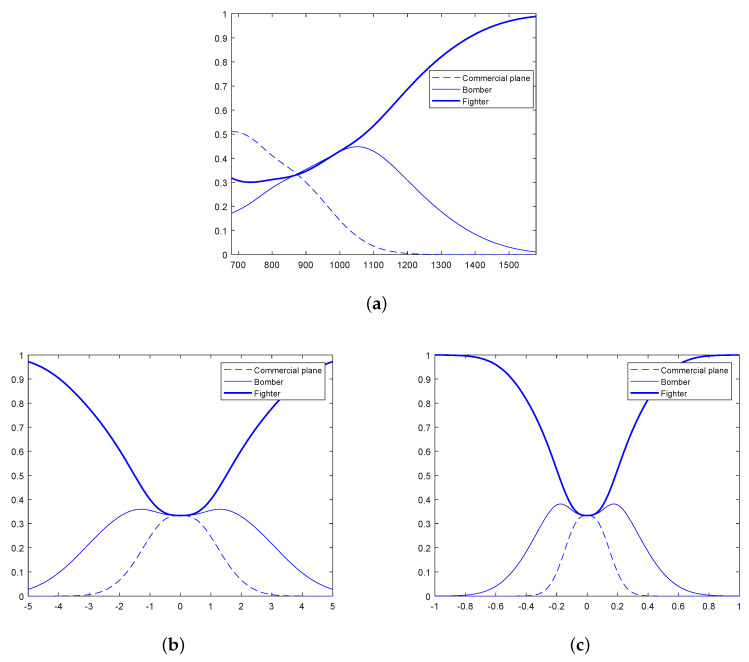
Prior pignistic class probabilities; (**a**) pignistic class probabilities for velocity, (**b**) pignistic class probabilities for acceleration, (**c**) pignistic class probabilities for turn rate.

In this section, simulations are performed to illustrate the effectiveness of the proposed algorithm. The scenarios under consideration contain several targets form three categories: ordinary targets, medium maneuvering target and high maneuvering targets, such as commercial plane, bomber and fighter, respectively. The model set consists of the constant velocity, constant acceleration and coordinated turn models. For the commercial plane, the transition probability of the dynamic models is 0.05, while the transition probabilities for the bomber and fighter are 0.1 and 0.2, respectively. The prior pignistic probability distributions of velocity, acceleration and turn rate for each category are Gaussian, i.e., $Betf∼N(\bar{x},\sigma )$, and the parameters are given in Table 1. In Figure 3, the pignistic probability functions of these classes are illustrated using thick, normal and dashed line, respectively.

In our method, different state feature subspaces are chosen for classification according to the dynamic modes. For CV, CA and CT models, the class beliefs are calculated based on the velocity, acceleration and turn angular rate, respectively. The performance of our algorithm is compared with the traditional methods in terms of the class probability.

**Figure 4:**
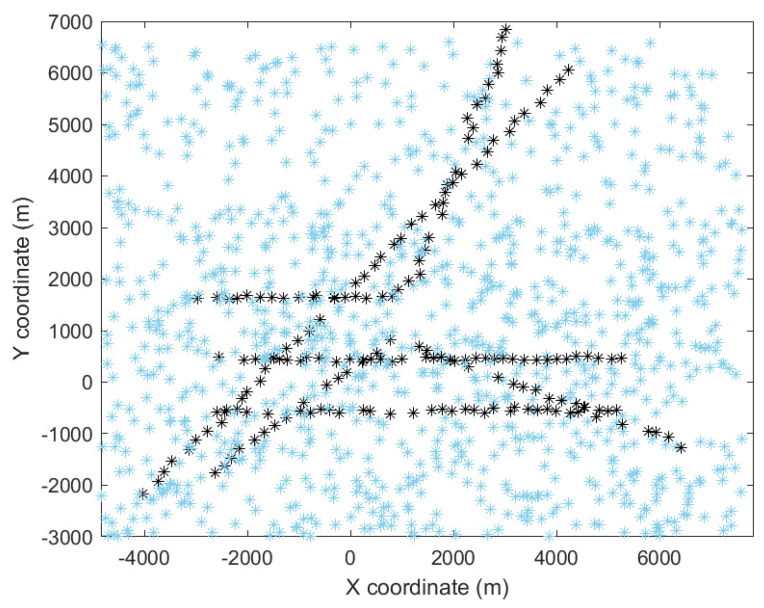
The tracks and measurements with miss detection and clutter.

In the scenario, five targets are involved, two are the high maneuvering targets such as the fighter, two other are the ordinary targets such as the normal commercial planes and the last one is a medium maneuvering target such as the bomber. These targets perform different maneuvers in (x,y) space. Target 1 appears at the beginning and firstly evolves with a constant velocity 870 km/h until 24 s, then executes a coordinated turn during 24 to 32 s at 0.35 rad/s. Finally, it performs a normal flight and disappears at 80 s. Target 2 appears at 8 s and disappears at the end, it firstly evolves with a low constant velocity at 850 km/h, then turns during 24 to 30 s with the turning angle rate 0.2 rad/s. Last, it speeds up to 1200 km/h within 8 s and escapes. Targets 3 and 4 appear from 16 s, and perform the uniform speed flight at 780 km/h throughout the process. Target 5 appears at 20 s and performs the uniform speed flight at 1080 km/h. The scenario is illustrated in Figure 4. The measurements of the tracks are drawn using black ‘*’, and the clutter are drawn using blue ‘*’.

In the simulation, the equation of the *i*th dynamic model is

(63) $$\begin{array}{c} x_{k}=F_{k,i}x_{k-1}+w_{k,i} \end{array}$$

where $F_{k,i}$ is the model-dependent state transition matrix, and $w_{k,i}$ is Gaussian noise with covariance $Q_{k,i}$. The constant velocity (CV) model has the following parameters

(64) $$\begin{array}{cc} F_{k,1} & =diag\left(\left[\begin{array}{ccc} 1 & T & 0\\ 0 & 1 & 0\\ 0 & 0 & 0 \end{array}\right],\left[\begin{array}{ccc} 1 & T & 0\\ 0 & 1 & 0\\ 0 & 0 & 0 \end{array}\right],1\right) \end{array}$$

(65) $$\begin{array}{cc} Q_{k,1} & =diag\left(\left[\begin{array}{ccc} T^{2} & T & 0\\ T & 1 & 0\\ 0 & 0 & 0 \end{array}\right]\sigma_{v}^{2},\left[\begin{array}{ccc} T^{2} & T & 0\\ T & 1 & 0\\ 0 & 0 & 0 \end{array}\right]\sigma_{v}^{2},0\right) \end{array}$$

where $\sigma_{v}$ is the process noise with covariance $\sigma_{v}^{2}=10\;m^{2}/s^{2}$. The parameters of CA model are

(66) $$\begin{array}{cc} F_{k,2} & =diag\left(\left[\begin{array}{ccc} 1 & T & \frac{1}{2}T^{2}\\ 0 & 1 & T\\ 0 & 0 & 1 \end{array}\right],\left[\begin{array}{ccc} 1 & T & \frac{1}{2}T^{2}\\ 0 & 1 & T\\ 0 & 0 & 1 \end{array}\right],1\right) \end{array}$$

(67) $$\begin{array}{cc} Q_{k,2} & =diag\;\left(\;\left[\begin{array}{ccc} \frac{1}{4}T^{4} & \frac{1}{2}T^{3} & \frac{1}{2}T^{2}\\ \frac{1}{2}T^{3} & T^{2} & T\\ \frac{1}{2}T^{2} & T & 1 \end{array}\right]\;\sigma_{a}^{2},\;\;\left[\begin{array}{ccc} \frac{1}{4}T^{4} & \frac{1}{2}T^{3} & \frac{1}{2}T^{2}\\ \frac{1}{2}T^{3} & T^{2} & T\\ \frac{1}{2}T^{2} & T & 1 \end{array}\right]\;\;\sigma_{a}^{2},\;0\;\right) \end{array}$$

where $\sigma_{a}$ is the process noise with covariance $\sigma_{a}^{2}=10$ m${}^{2}/$s${}^{4}$. The parameters of CT model are

(68) $$\begin{array}{cc} F_{k,3} & =\left[\begin{array}{ccccccc} 1 & \frac{sin(\omega T)}{\omega } & 0 & 0 & -\frac{1-cos(\omega T)}{\omega } & 0 & 0\\ 0 & cos(\omega T) & 0 & 0 & -sin(\omega T) & 0 & 0\\ 0 & 0 & 1 & 0 & 0 & 0 & 0\\ 0 & \frac{1-cos(\omega T)}{\omega } & 0 & 1 & \frac{sin(\omega T)}{\omega } & 0 & 0\\ 0 & sin(\omega T) & 0 & 0 & cos(\omega T) & 0 & 0\\ 0 & 0 & 0 & 0 & 0 & 1 & 0\\ 0 & 0 & 0 & 0 & 0 & 0 & 1 \end{array}\right] \end{array}$$

(69) $$\begin{array}{cc} Q_{k,3} & =diag\left(\left[\begin{array}{cc} \frac{T^{3}l_{1}}{3} & \frac{T^{2}l_{1}}{2}\\ \frac{T^{2}l_{1}}{2} & Tl_{1} \end{array}\right],0,\left[\begin{array}{cc} \frac{T^{3}l_{1}}{3} & \frac{T^{2}l_{1}}{2}\\ \frac{T^{2}l_{1}}{2} & Tl_{1} \end{array}\right],0,Tl_{2}\right) \end{array}$$

where $l_{1}$ and $l_{2}$ are the process noise with covariance $l_{1}=10^{-1}\;rad^{2}/s^{-4}$ and $l_{2}=10^{-4}\;rad^{2}/s^{-4}$, respectively.

The sensor receives range and bearing measurements

(70) $$\begin{array}{c} z_{k}=\left[\begin{array}{c} l_{k}\\ \theta_{k} \end{array}\right]+v_{k} \end{array}$$

where $l_{k}$ and $\theta_{k}$ are the true range and bearing measurement of a target that can be given by

(71) $$\begin{array}{ccc} l_{k} & = & \sqrt{{\left(p_{x}-s_{x}\right)}^{2}+{\left(p_{y}-s_{y}\right)}^{2}} \end{array}$$

(72) $$\begin{array}{ccc} \theta_{k} & = & \arctan\left[\frac{p_{x}-s_{x}}{p_{y}-s_{y}}\right] \end{array}$$

The position of the sensor $\left[p_{x},p_{y}\right]$ is located at [1000,−3000], and $v_{k}$ is the Gaussian measurement noise with the covariance $R_{k}=diag\left(\sigma_{r}^{2},\sigma_{\theta }^{2}\right)$, where $\sigma_{r}=5m$ and $\sigma_{\theta }=\left(\pi /180\right)rad$. The target detection probability is $p_{d}=0.92$, there are on average 35 clutters per scan within the multi-target moving area (clutter density is $2.08\times 10^{-7}$), and the sample time is 2 s. The target survival probability is $p_{s}=0.98$, and the target birth probability is $p_{b}=0.02$. The density of the new birth target is $b_{k}=N\left(x;m_{b};Q_{b}\right)$, where the parameters $m_{b}$ is equal to the position of the first measurement of each target, assume that the measurement in Cartesian coordinates is [x,y], the initial state of the birth targets is $m_{b}={\left[x,0,0,y,0,0,0\right]}^{\prime }$, while the state covariance is $Q_{b}=diag\left(100,10^{4},10,100,10^{4},10,0.1\right)$, respectively.

**Figure 5:**
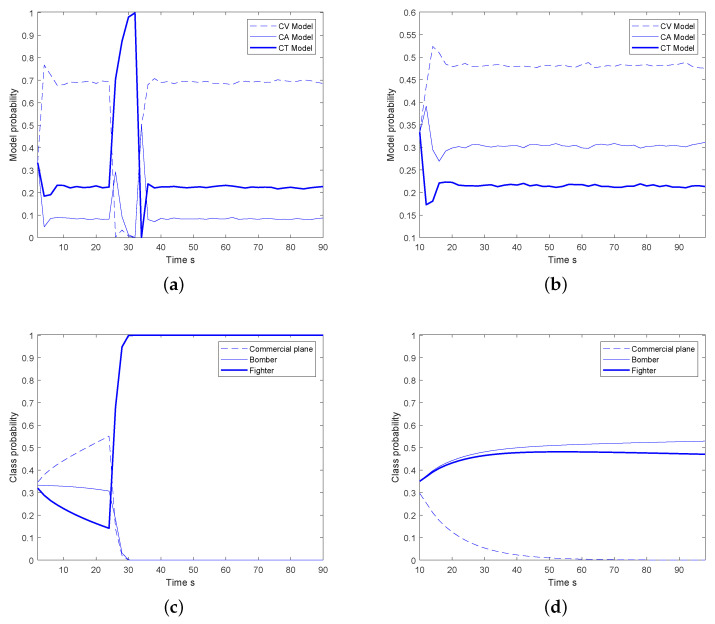
Classification results; (**a**) model probabilities of target 1, (**b**) model probabilities of target 5, (**c**) pignistic class probabilities of target 1, (**d**) pignistic class probabilities of target 5.

As shown in Figure 5a,b, take the targets 1 and 5 as example, the dynamic modes are correctly identified by the multi-model MGLMB filter in the case of dense clutter and miss detections. The model dependent class beliefs are calculated given corresponding dynamic state characteristics and then fused. The final classification results are illustrated in Figure 5c,d. For target 1, the pignistic class probability of commercial plane is the largest at first, the reason is that it performs a normal flight, and the classification result is mainly derived on basis of the velocity. When it turns, the pignistic probability of fighter rapidly increases because only the this kind of the target can do such sharp maneuver with high turning angular rate. For target 5, the object flies at a constant speed from beginning to the end, therefore, the classification results are derived using the velocity, the pignistic probabilities of fighter and bomber are approximately both equal to 1/2, while the commercial plane is excluded by the velocity. As a result that the kinematic characteristics are not very distinguishable, the target class cannot be effectively distinguished only from the speed, therefore the classification results still contain much uncertainty about the fighter and bomber.

**Figure 6:**
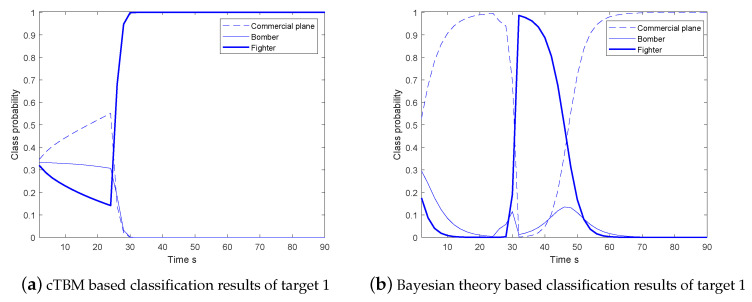
The comparison of the classification results of target 1; (**a**) continuous transferable belief model (cTBM), (**b**) Bayesian theory.

The comparison of the classification results for one target of the proposed MGLMB-cTBM algorithm and the Bayesian classifier is shown in Figure 6. Take the target 1 as example, before the target does a maneuver, the probability of normal commercial plane is larger using the Bayesian approach. In contrast, the classification results of the proposed MGLMB-cTBM algorithm still contain much uncertainties. When the target turns, the probability of the target being the fighter is the highest in both algorithms. After the target completes the maneuver, the probability of commercial plane obtained by the Bayesian classifier significantly increases again. The reason is that, the specific classification results are produces by the Bayesian classifier at each moment, and the prior probability of the commercial plane is larger under such speed. On contrary, for the classifier based on cTBM, the uncertainty of the target class is indicated using the confidence assigned to the focal element set $C=\{c_{1},c_{2},c_{1,2}\cdots \}$, and the reliabilities are translated into pignistic probabilities only when the decision is made. Therefore, when the evidences are insufficient, the difference among the pignistic class probabilities of different categories is not obvious. This result reflects the reasonability of the proposed MGLMB-cTBM algorithm.

**Figure 7:**
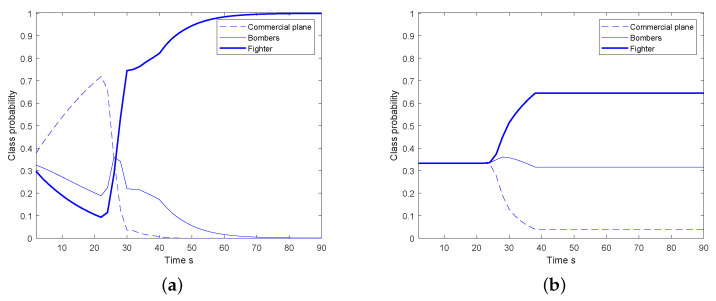
The comparison of the classification results of target 2; (**a**) Pignistic class probabilities of target 2 based on multiple models, (**b**) Pignistic class probabilities of target 2 based on single model.

In Figure 7, the superiority of the two level classifier is shown. Take the target 2 as example, as illustrated in the Figure 7a, the class probabilities of three classes are represented by the thick, normal and dash line, respectively. Before the target accelerates, the pignistic probability of commercial plane is larger because the classification results are derived mainly based on the velocity. After the medium maneuver performed during 24–32 s, the pignistic probabilities of bomber and fighter increase promptly. However, there still exists uncertainty about fighter and bomber, because all types of aircraft can do this maneuver except the commercial plane. Finally, the target class is determined on basis of the high velocity. The classification results of the method proposed in [7] are shown in Figure 7b, in which the target class is identified only based on the acceleration. It can be seen that, because the target makes medium maneuvering from 24 to 32s, the target class can be preliminary judged according to the acceleration, but no effective classification result is derived when the acceleration is 0 during the cruise section of flight. The kinematic characteristics are not fully utilized, and the acceleration is insufficient for distinguishing of the bomber and fighter absolutely. In contrast, our method identifies the motion mode firstly and classifies using appropriate dynamic characteristics, the target is finally identified according to the high speed. The classification performance is improved.

**Figure 8:**
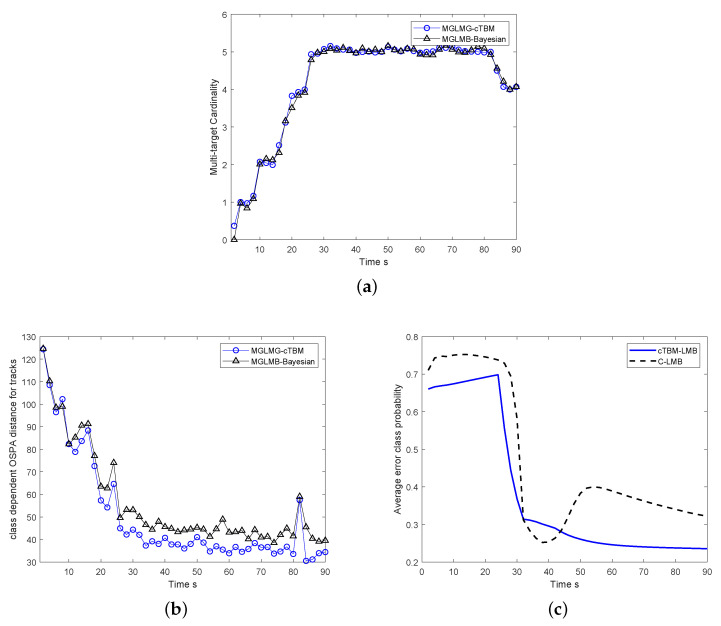
The comparison of tracking and classification results between multi-model marginal generalized labeled multi-Bernoulli (MGLMB)-Bayesian and MGLMB-cTBM; (**a**) cardinality estimates, (**b**) overall tracking performance, (**c**) average error class probability.

In Figure 8, the comparison of the overall detection, tracking and classification performance of 50 Monte Carol runs is illustrated. The proposed MGLMB-cTBM algorithm is compared with the MGLMB-Bayesian method in terms of the cardinality estimates, class dependent optimal subpattern assignment (OSPA) like distance for tracks, and average error class probability (the error between the classification results and the truth). In MGLMB-Bayesian method, the target class is introduced into conventional multi-model MGLMB filter, the posterior class probabilities and class dependent multi-target density are calculated based on the observation likelihood within Bayesian framework. As shown in Figure 8c, because the classification results derived within belief function framework is more reasonable and robust, the classification results of the cTBM based classifier is better. In Figure 8a,b, the detection and overall tracking performance of our proposed method is also better. The reason is that, according to more accurate classification results, appropriate dynamic modes are chosen during the estimation process, and the overall performance is improved. In Figure 8c The time scale represents the moving time of the targets. The proposed algorithm has an iterative implementation form, the motion state and class probability of each target are recursively calculated at each moment, and the number of iteration is actually equal to the time scan. The values of average error class probability change over time because the conditional beliefs are calculated at the current moment based on the dynamic state estimates, and then combined with the prior information using generalized Bayesian theorem. As more dynamic information of the target obtained, the class are more definite with the time recursion, and the values of average error class probability decreased over time.

To further evaluate the overall performance of the proposed algorithm, the algorithm works under 100 different scenarios that are randomly generated. Each scenario contains 5 targets moving within 90 s. The real class, appearing time, initial dynamic state and position of these targets are the same as in scenario 1. For the fighter, the beginning time of maneuver is random, and the probability of maneuvering is 0.02 at each moment during 10–80 s. The covariance of the Gaussian measurement noise $R_{k}=diag\left(200;5\times 10^{-3}\right)$, and there are average 50 clutters per scan within the multi-target moving area, both larger than scenario 1.

**Figure 9:**
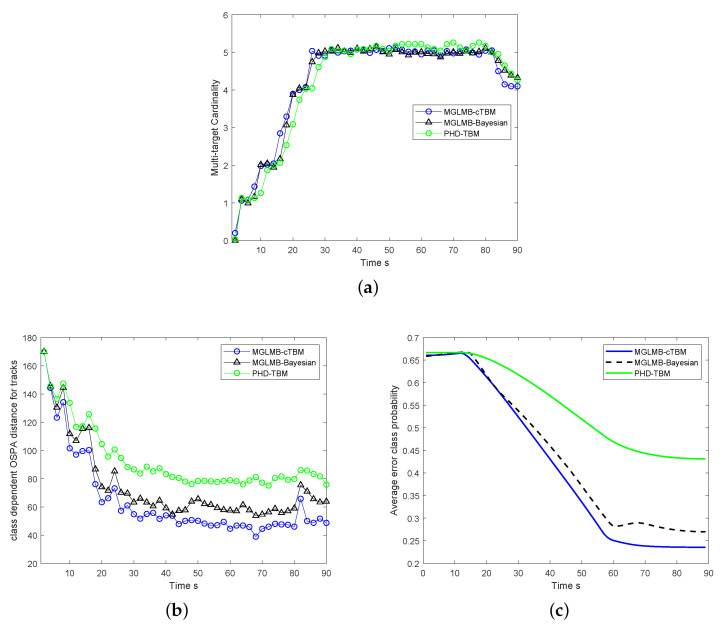
The comparison of tracking and classification results between MGLMB-Bayesian and MGLMB-cTBM; (**a**) cardinality estimates, (**b**) overall tracking performance, (**c**) average error class probability.

The proposed MGLMB-cTBM algorithm is compared with MGLMB-Bayesian and PHD-TBM algorithms [28] in terms of the cardinality estimates, overall tracking performance, and average error class probability. As illustrated in Figure 9a, although the cardinality estimates of all the algorithms converges to the truth, the estimates of PHD-TBM is worse within several scans after the target appears, the reason is that the MGLMB is an analytical multi-target tracker, and the dynamic state estimates is more accurate than PHD filter. In Figure 9c, the classification result of TBM-PHD is also worse because the results are derived only based on the dynamic modes, while the estimates are directly used for classification in cTBM based classifier. Moreover, both the classification and state estimation of MGLMB-cTBM algorithm is better than MGLMB-Bayesian method. Actually, the traditional method based on Bayesian framework is optimal in the usual sense, the performance of the proposed algorithm is better because it takes advantages of the belief function theory, the classification results derived within belief function framework is more reasonable and robust, as the target class is considered in state estimation, appropriate models are then used for multi-target tracking. As a result that the estimation and classification problems are jointly solved, more accurate classification and estimation results are both helpful to improve the overall performance.

The proposed MGLMB-cTBM algorithm is realized using Matlab and the open source tools. In the simulation, the density of each target is represented using 1000 particles. The operating environment of the program is Windows 10, i5 5200 Dual-core CPU 2.2 GHz, 8 GB RAM. The computational time of each step of MGLMB-cTBM, MGLMB-Bayesian and PHD-TBM methods is 15.7 s, 14.5 s and 0.095 s, respectively. The computational time of these algorithms is given in Table 2. As shown in the table, the computational time of PHD-TBM is far less than the other two methods, because the computational complexity of the PHD-based tracker is much lower than the MGLMB filter. As the target state estimates of MGLMB-cTBM need to be calculated given different classes and models, in addition, compared to the MGLMB-Bayesian filter, the beliefs of the particles need to be fused. Therefore, the computational time of MGLMB-cTBM algorithm is the largest. As illustrated in Figure 9, the estimation and classification results of MGLMB-cTBM are the best, therefore, the increase in the amount of the computation leads to an improvement in the performance. Actually, the Gaussian mixture implementation can be used under linear Gaussian condition, and the computational time of MGLMB-cTBM algorithm can be further reduced.

## 5. Conclusions

This paper presents a new solution to multi-target joint detection, tracking and classification with target number and observation uncertainty based on the marginal GLMB filter and cTBM. Explicit state estimates and pignistic class probabilities of each target are produced by the proposed algorithm, and the particle implementation is also given. For multi-target tracking, a class dependent multi-model MGLMB filter is developed to calculate the target existing probabilities, dynamic model probabilities and model-dependent target state estimates. For target classification, a two-level classifier is designed, the class beliefs are first computed on corresponding state feature subspace according to different models, and then fused to derive the final results. Numerical examples are also performed to demonstrate the effectiveness and robustness of the proposed algorithm. As both the dynamic modes and the state estimates are considered by the classifier, the kinematic characteristics are fully utilized to improve the classification performance. Moreover, because the explicit class piginistic probability is only computed when the decision is made, the uncertainty of the class is well described using the belief functions during the recursion, and the classification results are more reasonable. Actually, the designed classifier extracts more information from the measurement and kinematic states to adaptively improve the tracking performance by choosing the proper motion modes, etc. In addition, because the estimation and classification problems are jointly solved, the performance of the detection, tracking and classification are further improved compared with the traditional methods.

## Figures and Tables

**Table 1:** Numerical values for priori pignistic class probability density.

	Commercial Plane	Bomber	Fighter
Velocity interval (km/s)	[680, 1020]	[850, 1190]	[720, 1580]
Velocity var (km/s)	120	160	250
Accelerate mean (g)	0	0	0
Accelerate var (g)	0.8	1.2	1.6
Turn rate mean (rad/s)	0	0	0
Turn rate var (rad/s)	0.1	0.2	0.4

**Table 2:** Average computational time.

MGLMB-cTBM	MGLMB-Bayesian	PHD-TBM
15.7 s	14.5 s	0.095 s
